# Synthesis and Micromechanistic Studies of Sensitized Bentonite for Methyl Orange and Rhodamine-B Adsorption from Wastewater: Experimental and DFT-Based Analysis

**DOI:** 10.3390/molecules27175567

**Published:** 2022-08-29

**Authors:** Sadaf Mutahir, Tayyaba Irfan, Nimra Nadeem, Muhammad Humayun, Muhammad Asim Khan, Moamen S. Refat, Chundong Wang, Tahir Ali Sheikh

**Affiliations:** 1 Department of Chemistry, University of Sialkot, Sialkot 51300, Pakistan; 2 School of Chemistry and Chemical Engineering, Linyi University, Linyi 276000, China; 3 Wuhan National Laboratory for Optoelectronics, School of Optical and Electronics Information, Huazhong University of Science and Technology, Wuhan 430074, China; 4 Department of Chemistry, College of Science, Taif University, P.O. Box 11099, Taif 21944, Saudi Arabia; 5 Institute of Chemistry, The Islamia University of Bahawalpur, Baghdad-ul-Jadeed Campus, Bahawalpur 63100, Pakistan

**Keywords:** wastewater treatment, simulations, refractory pollutants, Langmuir and Freundlich, kinetic studies

## Abstract

This work reports the formation of a novel adsorbent, prepared by activating bentonite with cinnamic acid, which is highly efficient to remove dyes from wastewater. The adsorption efficiency of the cinnamic acid activated bentonite was compared with unmodified bentonite by removing methyl orange and rhodamine-B from polluted water. The characterization was performed through X-ray diffraction (XRD) Fourier transform infrared (FTIR) and scanning electron microscopy (SEM). The results indicated that acidic pH and low temperature were more suitable for the selected dyes adsorption. The analysis of the data was done by the Langmuir and Freundlich isotherms; the Freundlich isotherm showed more suitability for the equilibrium data. The data were further analyzed by pseudo-first and pseudo-second-order models to study adsorption kinetics. The results showed that methyl orange and rhodamine-B adsorption obeyed pseudo-order kinetics. The results obtained from this research suggested that acid activation of bentonite with cinnamic acid increased the surface area of the clay and hence enhanced its adsorption efficiency. The maximum adsorption efficiency for the removal of methyl orange and rhodamine-B was up to 99.3 mg g^−1^ and 44.7 mg g^−1^, respectively, at 25 °C. This research provides an economical modification technique of bentonite, which makes it cost-effective and a good adsorbent for wastewater treatment.

## 1. Introduction

In recent times, industries have been growing day by day and the increase in the number of industries has caused many harmful aspects. One of these is water pollution, which is a very serious issue and dangerous for living organisms, because there is no proper disposal system in different areas. Therefore, an efficient adsorbent is required to remove contaminants from wastewaters [1]. Polluted water is not only affecting human beings but animals and plants as well. Industries use different chemicals for the production of their products, and then these chemicals are released as effluents through wastewater which is directly discharged into the ponds, lakes, rivers, and seas [2,3,4]. The effluents released contain chemical pollutants, heavy metals, oils, grease, and dyes. Organic pollutants are biodegradable so water polluted with them can be easily purified but inorganic pollutants are nonbiodegradable, so water polluted with them is difficult to treat. Plants are being destroyed by polluted water, they uptake pollutants including dyes when grown in a polluted environment, and metals also get stored in the tissues of humans and other species [5]. The contaminants can also infiltrate groundwater aquifers and affect underground water [6]. Due to the growing pace of population and development in most nations throughout the world, wastewater treatment is critical to meet water consumption for industries, agriculture, and home applications [7,8].

Every year, more than 700,000 tons of dyes are produced and 60–70% of these dyes are azo dyes. The usage of dyes is expanding and is mostly utilized in industries, causing an excessive quantity of dyes to stay in raw form during the dying and later going into wastewaters and remaining suspended in the effluent. Water restoration is critical for ensuring a long-term supply of clean water to fulfill the rising demand for clean water for future industrial development and growing populations [9]. The primary goal of dye synthesis is to produce a color that is strong, stable, and that does not fade over time or in the sunshine. As a result, most dyes have complicated structures and an aromatic ring that is difficult to break. If dyes are disposed of directly into water reservoirs, they are hazardous, cannot be recycled, and create a rise in the concentration of contaminants [10]. Eventually, the amount of pollution reaches critical levels, preventing the restoration of a microbial community, and the quality of water deteriorates permanently. [11]. Toxic dyes can cause reproductive disorders, kidney diseases, dermatitis, allergy, liver, and cancer. The treatment of wastewater demands economical techniques that can produce water of high quality for reprocessing [12]. The recycling of wastewater might decrease the requirement for freshwater. Recently, there has been an increase in the usage of recycled water in many industries. By increasing the biochemical as well as chemical oxygen requirement, textile dyes primarily have an impact on the beautiful appearance of aquatic creatures. Furthermore, it reduces photosynthesis, obstructs plant development, and enters the food chain. The potential for bioaccumulation encourages toxicity, carcinogenicity, and mutagenicity.

There are various procedures to remove dyes and other pollutants from contaminated water. These methods include coagulation/flocculation, chemical precipitation, reverse osmosis, electrodialysis, and adsorption [13,14,15]. The most used and preferred technique is adsorption. In this technique, liquid and gaseous contaminants are adsorbed on the solid adsorbent and form an adsorbate. The contaminant particles stick to the surface by chemical or physical adsorption [16]. Different adsorbents are used such as commercial zeolites, silica gel, limestone, dolomite, chitosan, bentonite, lignin, and activated carbon. This process is used commercially for the removal of color, traces of organics, and toxic metals [17]. The only limitation of this technique is that low-cost adsorbents are not easily available [18]. Adsorption is a simple procedure that can be applied for the removal of colors from wastewater. It can degrade physiologically and chemically stable substances [19,20]. The adsorption of colors from water is affected by parameters such as the initial amount of dye, contact time, and temperature. Adsorption isotherms are used to achieve a balance between the quantity of adsorbed colors on the adsorbent and the quantity of adsorbed colors on the adsorbent. The isotherms of Freundlich and Langmuir are commonly employed to investigate adsorption data. To assess the dynamics of adsorption, printed first-order and second-order concealed dynamic patterns are used [21].

Natural clays have gained popularity as cost-effective adsorbents in recent years because they are readily available and may be activated to enhance their surface area, capacity to adsorb, and application range. Bentonite has proved to be an efficient adsorbent to remove dyes. Because of the negative charge on the surface of bentonite, it can only be used to remove basic colors. Furthermore, natural bentonite’s adsorption capacity is limited due to its small surface area [22]. These points have prompted researchers to modify bentonite to boost its adsorption capacity. Bentonites with a modified surface have a high potential to operate as a substitute for activated carbon, which is widely employed. To increase the adsorption capabilities of bentonite clay, a variety of approaches are being investigated, including acid activation and heat treatment, cationic surfactant treatment, and polymer modification [23]. Bentonite is a clay that contains phyllo-silicate layers, it has sheets that are octahedral and tetrahedral containing a ratio of 2:1, and it is unique due to its composition and its structural importance [24]. It has mesopores, macropores, and micropores in its structure, on which adsorption depends. Bentonite has different applications in water purification, pharmaceutical preparations, nondrip paints, fertilizer sprays, and drilling fluids [25]. Bentonite is a good adsorbent due to different advantages that include a high cation change capacity, high porosity, large surface-to-area ratio, and being economical. It needs modification for the adsorption of dyes [26]. The modification increases the surface area and efficiency of the clay by increasing its adsorption capacity. Clays can be activated with acids to modify their properties, which includes increasing surface area and average pore volume [27]. The chemical properties of clay, that is, the ability to exchange cation and surface acidity, can be altered, resulting in the parameters needed for an efficient adsorbent. Acid modification is a good approach for enhancing surface area so that the breakdown of the structure of crystals can be monitored [28].

To the best of our knowledge, no study has reported the preparation and application of cinnamic acid modified bentonite for the removal of methyl orange from wastewater. For this purpose, the synthesis and characterization of cinnamic acid modified bentonite were performed for the removal of MO and RhB from polluted water. This study also included an analysis based on density functional theory.

## 2. Results

### 2.1. Material Characterization

**Figure 1:**
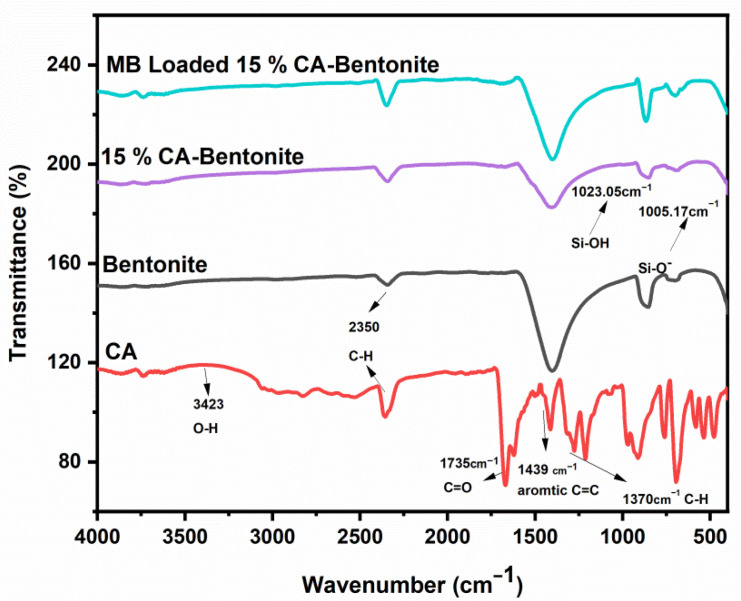
FTIR spectrum of raw and cinnamic acid activated bentonite clay.

Figure 1 shows the Fourier transform infrared spectroscopy spectrum. The bands at 3596.5 cm^−1^ are allocated to the OH stretching mode for clays observed from the IR table [29]. These coupled OH (hydroxyl groups) are characteristic of bentonite clay. The OH band at 3595.5 cm^−1^ is located in such a way that it permits the hydrogen bonding to oxygens of the nearby layers [30]. The dipoles of this OH group are perpendicular to the crystal surface. It gives a negative charge to the overall clay surface. The bands at 1023.05 cm^−1^ and 1005.17 cm^−1^ show the bending of Si-OH and Si-O^−^, respectively [31]. The most prominent difference between the peaks of raw and activated bentonite is observed around about 800 cm^−1^ and 1400 cm^−1^, which shows the change in the structure of bentonite due to acid attack.

**Figure 2:**
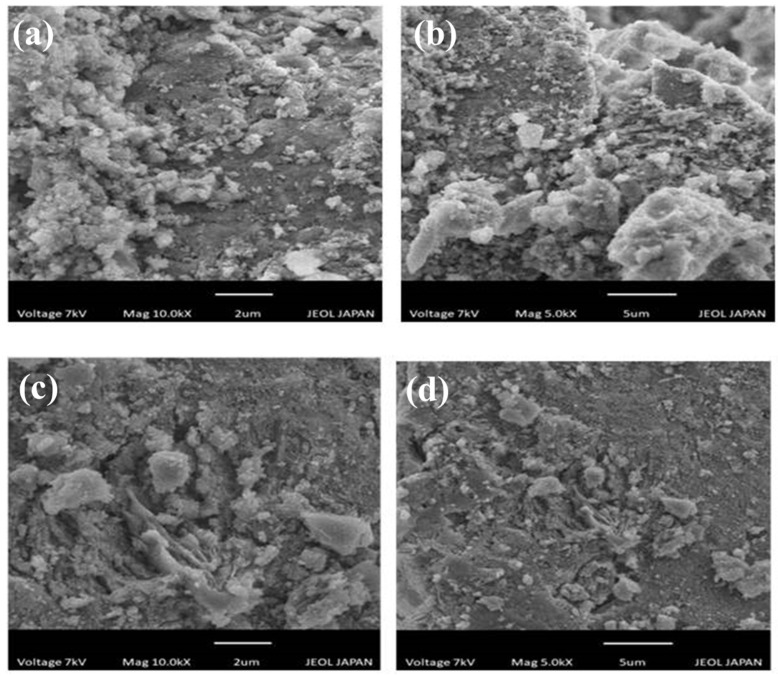
SEM images of (**a**,**b**) raw bentonite and (**c**,**d**) cinnamic acid modified bentonite.

Figure 2 shows the scanning electron microscopy images of raw and cinnamic acid modified bentonite for the analysis of the changes in the structure of bentonite after acid activation. Acid activation causes the cations to leach, which creates spaces in the structure of bentonite and makes it more porous. Figure 2a,b show the raw bentonite in which there are clumps of the uneven surface that are less porous. Figure 2c,d show cinnamic acid modified bentonite where the surface is highly porous and even [32].

**Figure 3:**
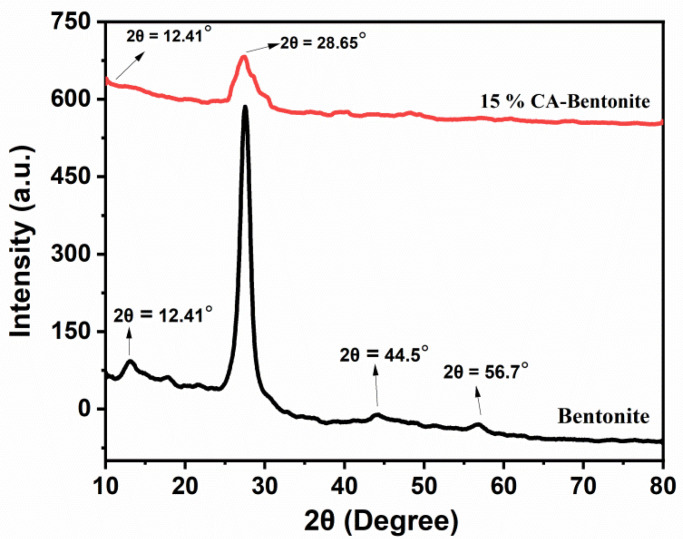
XRD spectrum of raw and cinnamic acid modified bentonite clay.

Figure 3 displays the XRD spectrum, which shows that the clay is highly crystalline because the peaks are narrow. The main peak at 2θ = 28.65° is the characteristic band for quartz (SiO_2_). Quartz is a silica polymorph that consists of interrelated tetrahedral which make a strong three-dimensional network. The peaks at 2θ = 12.41°, 44.5°, and 56.7° are characteristic of bentonite clay. Figure 3 shows that the montmorillonite section of the clay changed after acid activation at 12.41°. Furthermore, the interlayer spacing of the acid-activated bentonite decreased, which indicates a deformation of the crystal structure.

**Figure 4:**
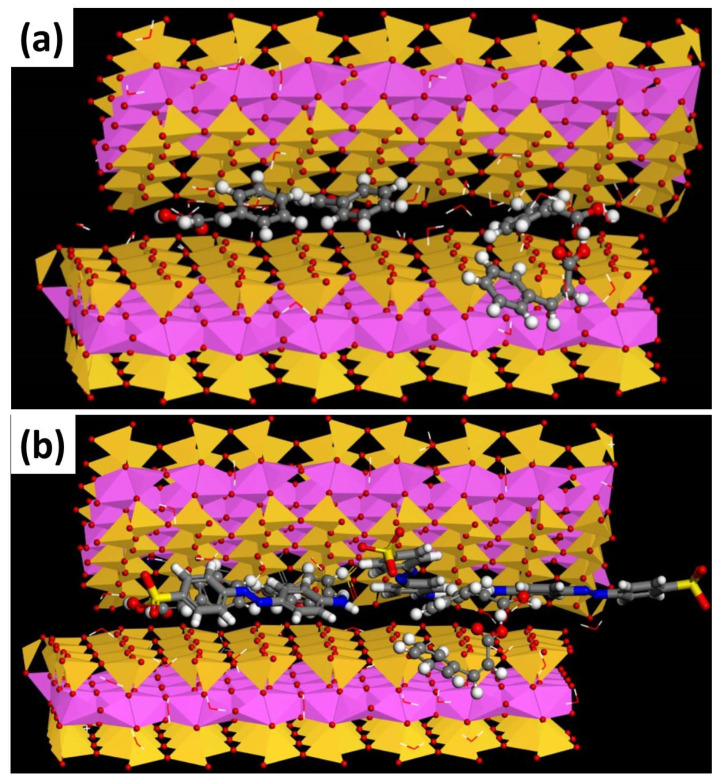
Schematic representation of CA modified bentonite (**a**) and MO adsorbed, CA modified bentonite (**b**), after equilibrium, a snapshot of the adsorption system (Note: C = grey, N = blue, H = white, O = red, Si = yellow, Al = pink, Mg = green for all species).

Material Studio 6.0 (Accelrys, San Diego, CA, USA) software was used to describe the probable interlayer structures of CA modified bentonite with 15% loading levels of cinnamic acid, including the interlayer of water box. To explore the interaction of the cinnamic acid and CA modified bentonite, a molecular simulation was run using the Adsorption locator and Forcite modules of Materials Studio. The C2/m space group was used to build the CA modified bentonite model. The supercell was produced with the parameters a = 0.523 nm, b = 0.906, c = 1.230 nm, α = γ = 90°, β = 99°, and the dimensions were 4a × 2b × 1c. Water was introduced into the interlayer. Four numbers of cinnamic acid molecules were added to the layer depending on the cation exchange capacity. With the least amount of energy, the best arrangement was found. Figure 4 depicts the probable interlayer structures with CA modified bentonite (a) and MO adsorbed, CA modified bentonite (b), including the interlayer of water [33].

### 2.2. Effect of Contact Time

**Figure 5:**
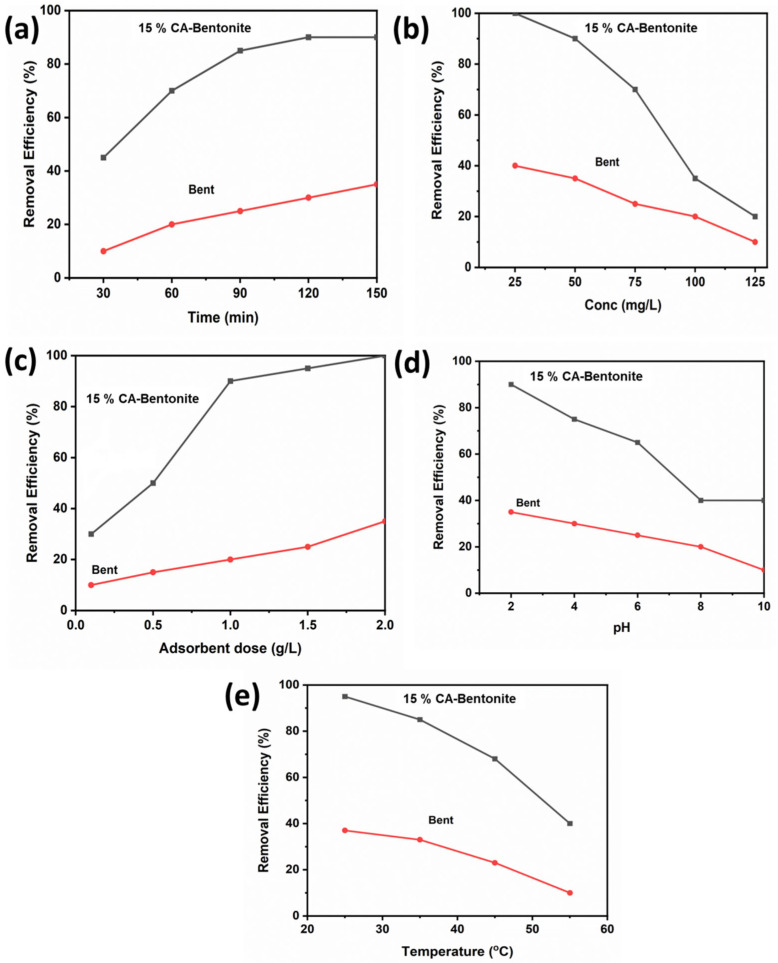
Effect of contact time (**a**), initial concentration of dye (**b**), adsorbent dose (**c**), pH (**d**), and temperature (**e**), on methyl orange adsorption on cinnamic acid modified bentonite and on raw bentonite.

The influence of contact time on the adsorption of dye was investigated, and 1 g of modified and unmodified bentonite was added separately to a 200 mL solution of dye having a concentration of 50 mg/L. The flasks were placed on a rotatory shaker for different time intervals ranging from 30 min to 120 min. The flasks were removed from the rotatory shaker after the known period. The concentration of the dye in supernatant liquid was measured by using a UV–vis spectrophotometer. We noted that the removal of methyl orange was increased by increasing the contact time. The maximum adsorption was achieved in 2 h; this higher adsorption was achieved due to the availability of more empty spaces as compared to unmodified bentonite (Figure 5a) [34].

### 2.3. Effect of Initial Dye Concentration

To study the influence of the initial concentration of dye on adsorption by using unmodified and acid-modified bentonite, the equilibrium data were obtained. The concentration range used to study the effect of the initial concentration of dye was from 25 mg/L to 125 mg/L. Briefly, 1 g of adsorbent was added to 200 mL of dye solution. Flasks were kept on the rotatory shaker for 2 h. After this, the solutions were filtered. The dye concentration of supernatant liquid was calculated by using a UV–vis spectrophotometer. The removal rate was higher at the early stage because there were more empty spaces, which increased adsorption. At equilibrium conditions, up to 50 mg/L concentration of dye could be best removed (Figure 5b) [35].

### 2.4. Effect of Adsorbent Dosage

The dosage of the adsorbent ranged from 0.1 g to 2 g. A calculated amount of acid-modified bentonite was added to 200 mL of dye solution of 50 mg/L concentration. The flasks were placed on a rotatory shaker for 2 h. After this, the solutions were filtered. The dye concentration of supernatant liquid was measured by using a UV–vis spectrophotometer. It was observed that adsorption was increased by increasing the adsorbent dosage, but after 2 g of adsorbent dose, the removal rate remained the same with no further improvement. By increasing the adsorbent dose, more empty spaces were available due to an increased surface area and the adsorption rate increased (Figure 5c) [36].

### 2.5. Effect of PH

The influence of pH on dye adsorption by acid-modified bentonite was investigated. The pH of the methyl orange solution was adjusted by 0.1 M HCl and 0.1M NaOH. A weighed amount of acid-modified bentonite was added to dye solutions of different pH. The pH of the initial solution dye was maintained at 2, 4, 6, 8, and 10. The samples were kept in a rotatory shaker for 2 h. After this, the solutions were filtered. The concentration of dye in supernatant liquid was calculated by using a UV–vis spectrophotometer. The removal was best in acidic conditions because methyl orange is an anionic dye and in alkaline conditions, the hydroxyl groups compete with the methyl orange to get adsorbed on the adsorbent (Figure 5d) [37].

### 2.6. Effect of Temperature

The effect of dye adsorption on acid-modified bentonite was studied at various temperatures of 25, 35, 45, and 55 °C. A weighed amount of acid-modified bentonite was added to a 200 mL dye solution at different temperatures. The samples were kept on the rotatory shaker for 2 h. After this, the solutions were filtered. The dye concentration of supernatant liquid was calculated by using a UV–vis spectrophotometer. Low temperature is favorable for methyl orange adsorption because at higher temperatures van der Waals forces and hydrogen bonds are weak, and thus, the interaction between methyl orange and bentonite is poor (Figure 5e) [38].

### 2.7. Adsorption Isotherms

**Figure 6:**
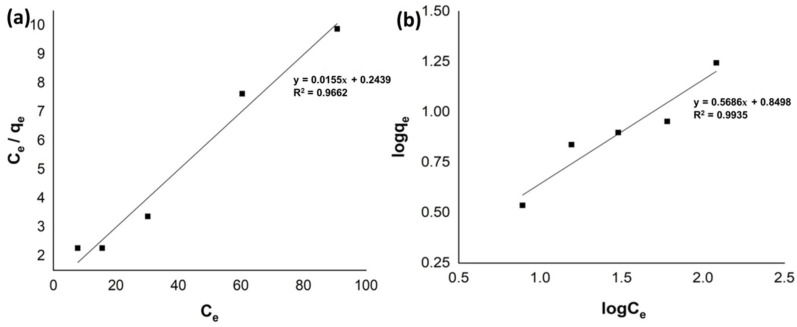
Langmuir (**a**) and Freundlich (**b**) adsorption isotherms for methyl orange adsorption.

The adsorption mechanism can be calculated by examining the adsorption data. Adsorption isotherms can establish an equilibrium between different amounts of the dyes that get adsorbed on the adsorbent. Langmuir and Freundlich’s isotherms were applied here to investigate the adsorption data (Figure 6). The primary supposition of the Langmuir model is that a monolayer forms on the adsorbent’s surface, meaning that a single dye molecule may be adsorbed on one adsorption site and that forces between molecules diminish with distance [39]. It was also expected that the surface of the adsorbent would be homogenous and have comparable and energetically similar adsorption sites (Figure 6a) [40].

The isotherm fitted the adsorption data because it was linear throughout the concentration range.

Freundlich’s isotherm model is suitable for heterogeneous systems, and it indicated the formation of multilayers (Figure 6b) [41]. The Langmuir and Freundlich models’ correlation coefficients were 0.9662 and 0.9935, respectively. The high value of the correlation coefficient R^2^ in the Freundlich model shows that this model is more suitable for the adsorption process of methyl orange.

### 2.8. Adsorption Kinetics

**Figure 7:**
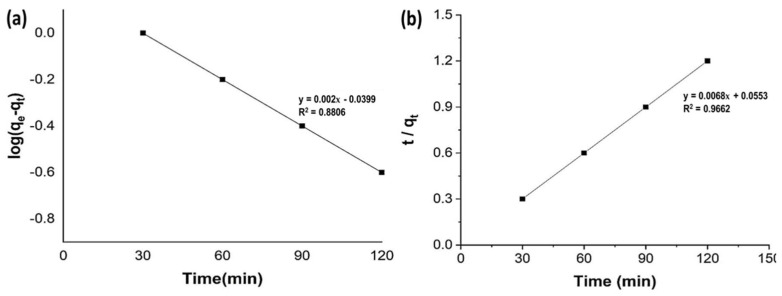
Pseudo-first- (**a**) and pseudo-second- (**b**) order kinetic model for methyl orange adsorption.

The kinetics of adsorption of methyl orange on unmodified and acid-activated bentonite were investigated. Pseudo-first-order and pseudo-second-order kinetics were used to examine experimental data (Figure 7). The pseudo-first-order model of kinetics is widely used to determine the kinetic behavior of a system (Figure 7a) [42,43].

The assumption on which the pseudo-second-order model depends is that chemisorption may be the rate-limiting step, which includes the exchanging or sharing of electrons between adsorbate and adsorbent by involving valence forces. The pseudo-second-order model shows the maximum adsorption capacity values and these results have been reported by other researchers [40]. Our results showed that methyl orange adsorption followed the pseudo-second-order model (Figure 7b). The pseudo-first- and second-order constants parameters were 0.033 (k_1_), 0.8806 (R^2^) and 0.0061 (k_2_), 0.9662 (R^2^), respectively.

### 2.9. DFT-Based Analysis of Adsorption Mechanism

**Figure 8:**
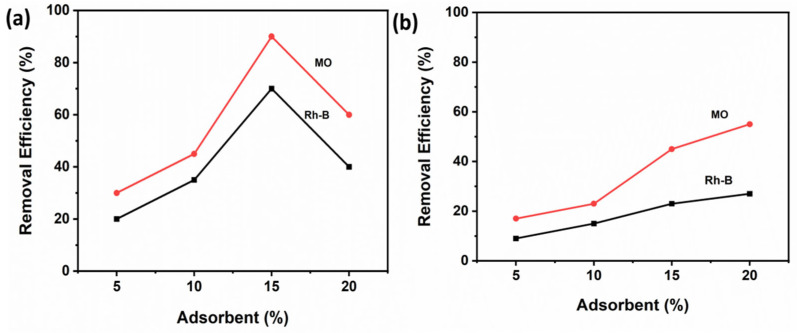
Adsorption efficiency of methyl orange and Rh-B on cinnamic acid modified bentonite (**a**) and on raw bentonite (**b**).

**Figure 9:**
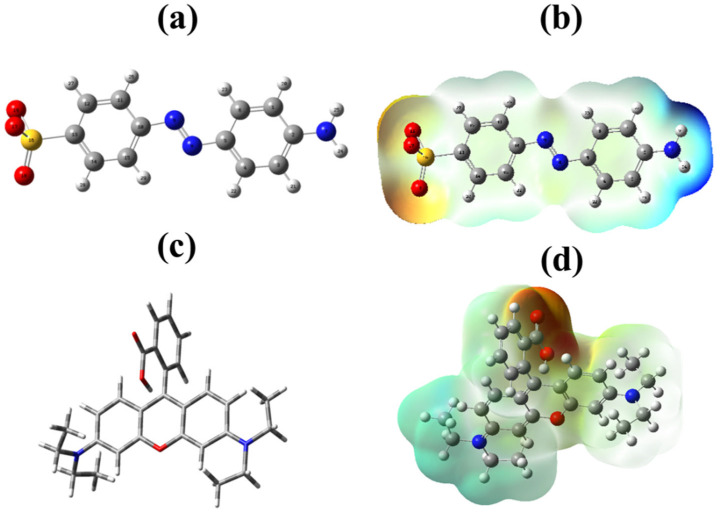
(**a**) Optimized structure of methyl orange, (**b**) MEP of methyl orange, (**c**) optimized structure of rhodamine-B, and (**d**) MEP of rhodamine-B.

Cinnamic acid modified bentonite was successfully used to remove methyl orange and Rh-B from wastewaters; for comparison, unmodified bentonite was used (Figure 8). To investigate the adsorption mechanism, density functional theory (DFT) is among the most popular techniques available in computational chemistry. It is used to investigate the electronic structures of atoms, molecules, and solids. To investigate possible molecular interactions between modified bentonite and methyl orange, DFT calculations were done by using a basis set of 3-21G B3YLP level theory. The optimized structures of methyl orange and rhodamine-B are shown in Figure 9a,c.

The MEP diagram shows that methyl orange and rhodamine-B have reactive sites (Figure 9b,d). The blue region shows a higher potential, and the red region shows a lower potential. The nucleophile attacks the blue region while the electrophile attacks the red region [44]. The *O* atom of the C = *O* group of cinnamic acid can be a possible site for an electrophilic attack and the *O* atom of the OH group of the bentonite can be a possible site for a nucleophilic attack. The electrophilic interaction will occur at the oxygen of the carbonyl group of cinnamate, and the nucleophilic interaction will occur at the hydroxyl group of the bentonite [45].

## 3. Materials and Methods

### 3.1. Reagents and Chemicals

The sodium bentonite used in this study was purchased from H.A scientific trader Faisalabad (Faisalabad, Pakistan). Anionic dye methyl orange, cationic dye rhodamine-B, cinnamic acid, hydrochloric acid, and sodium hydroxide were purchased from Sigma Aldrich (Beijing, China) and were of analytical grade.

### 3.2. Synthesis of Cinnamic Acid Modified Bentonite

The bentonite was washed with distilled water by stirring it for 48 h and then it was allowed to settle for 24 h. The upper layer of water was decanted, and then the slurry of the clay was poured onto a filter paper and then rinsed again with distilled water till the filtrate became colorless. The filtered slurry of clay was then dried in an oven at 60 °C for 24 h. After drying, the clay was crushed in a pestle and mortar to get a fine powder of the clay. The obtained powdered clay was then stored for further modification. To modify clay with cinnamic acid, the solution of cinnamic acid was made by dissolving cinnamic acid crystals in distilled water and a sodium hydroxide solution. The solution of cinnamic acid was stirred until it became clear. Then, a weighed amount of bentonite was added to different flasks of cinnamic acid solution of different concentrations to make solutions of different percentages (5%, 10%, 15%, and 20%). The solutions of different percentages were then stirred for 2 h at 90 °C. After stirring, the solutions were filtered and then washed several times with distilled water. The slurry was then dried in the oven at 60 °C for 48 h. The dried and crushed cinnamic acid modified bentonite powder was then placed in an airtight container for further experiments [7].

### 3.3. Characterization Method

The effects of the acid activation on the characteristics of clay were examined by using different characterization techniques: XRD, FTIR, and SEM. X-ray diffraction was performed to identify the crystalline structure and chemical composition of the sample. FTIR was performed to derive information about the functional groups and structural changes of the bentonite upon acid activation. The SEM analysis was used to examine the morphology, composition, and other physicochemical properties of clay before and after modification.

### 3.4. Adsorption Experiments

The adsorption of methyl orange on unmodified and modified bentonite was investigated by using different conditions. Different concentrations of the cinnamic acid modified bentonite were used for adsorption of methyl orange and rhodamine-B. The dye solutions of different concentrations were prepared by mixing weighed amount of methyl orange and rhodamine-B in distilled water. Briefly, 0.1 g, 0.5 g, 1 g, 1.5 g, and 2 g of modified bentonites were added in separate flasks. Then, 200mL of dye solution of concentrations 25 mg/L, 50 mg/L, 75 mg/L, 100 mg/L, and 125 mg/L was added in each flask. The flasks were placed in the water bath at an orbital shaker for 2 h. After this, the solutions containing dye and modified bentonite were filtered and the filtrate was stored in vials for further studies. Different factors including initial dye concentration, adsorbent dose, pH, and temperature were studied. To study the effect of pH, 0.1 M HCl and NaOH were used to adjust the pH of the solution. The temperature was also varied to study its influence on adsorption. The concentration of dye in the solution was calculated by a UV–vis spectrophotometer.

### 3.5. Model Parameters

Langmuir (Equation (1)) and Freundlich (Equation (2)) adsorption models were applied for the analysis of the data. They are represented by the following equations:

(1) $$\frac{\mathrm{Ce}}{\mathrm{Qe}}=\frac{1}{K_{L}q_{m}}+\frac{1}{q_{m}}\mathrm{Ce}$$

(2) $${\mathrm{lnq}}_{e}=\frac{1}{n}\mathrm{lnCe}\;+{\;\mathrm{lnK}}_{F}$$

C_e_ is the concentration of the dye at equilibrium (mg/L), q_e_ is the amount of adsorbed dye per unit of bentonite (mg), q_m_ is the Langmuir constant for capacity of adsorption (mg/g), and K_L_ is the Langmuir constant for adsorption energy (L/g). k_f_ and n are Freundlich constants. Pseudo-first-order (Equation (3)) and pseudo-second-order (Equation (4)) kinetic models were applied to explain the kinetics of methyl orange adsorption on cinnamic acid modified bentonite.

(3) $$\ln`\left(\frac{q_{e}}{q_{e-q_{t}}}\right)=\frac{K_{1}t}{2.303}$$

K_1_ (1/min) is the adsorption rate constant for the pseudo-first order while q_t_ and q_e_ are the adsorption capacities at time t and at equilibrium.

The pseudo-second-order kinetic model is given by Equation (4).

(4) $$\frac{t}{q_{t}}=\frac{t}{q_{e}}+\frac{1}{K_{2}q_{e}^{2}}$$

K_2_ is a constant of the pseudo-second order, and q_t_ (mg/g) and q_e_ (mg/g) are adsorption capacities at time t and equilibrium, respectively.

## 4. Conclusions

In conclusion, we developed a novel cinnamic acid modified bentonite material for the adsorption of methyl orange and rhodamine-B from wastewater. The results showed that cinnamic acid modified bentonite had a higher adsorption capacity as compared to pristine bentonite. The adsorption was more favorable at acidic pH and low temperature. Freundlich’s isotherm had a better suitability towards the adsorption data, which indicated that methyl orange and rhodamine-B formed a multilayer on the adsorbent instead of a monolayer. The kinetics of methyl orange adsorption on the adsorbent was examined by pseudo-first-order and pseudo-second-order kinetics. The most suitable correlation coefficient was obtained by the pseudo-second-order kinetics. The maximum adsorption efficiency for the removal of methyl orange and rhodamine-B was up to 99.3 mg g^−1^ and 44.7 mg g^−1^, respectively at 25 °C. Furthermore, cinnamic acid modified bentonite can replace costly adsorbents, such as activated carbon, because bentonite is a low-cost adsorbent and easily available. Modifying bentonite with cinnamic acid is an economical method, and it increases the adsorption capacity, so it will help in the future to replace expensive adsorbents for dye removal.

## Data Availability

Not applicable.

## Sample Availability

Not available.
